# Dementia Severity Is Associated With Early Potentially Avoidable Readmissions in an Acute Care Hospital

**DOI:** 10.1093/geroni/igaa057.252

**Published:** 2020-12-16

**Authors:** Seigo Mitsutake, Tatsuro Ishizaki, Rumiko Tsuchiya-Ito, Akira Hatakeyama, Mika Sugiyama, Ko Furuta, Kenji Toba, Hideo Ito

**Affiliations:** 1 Tokyo Metropolitan Institute of Gerontology, Itabashi-ku, Tokyo, Japan; 2 Dia foundation for research on Ageing Societies, Tokyo, Japan; 3 Tokyo Metropolitan Geriatric Hospital, Itabashi-ku, Japan; 4 Tokyo Metropolitan Institute of Gerontology, Tokyo, Tokyo, Japan; 5 Tokyo Metropolitan Geriatric Hospital, Itabashi-ku, Tokyo, Japan; 6 Tokyo Metropolitan Geriatric Hospital and Institute of Gerontology, Itabashi-ku, Tokyo, Japan

## Abstract

Understanding the association of dementia severity with early potentially avoidable readmissions (PAR) could encourage the identification of the target patients for the health care providers to provide transitional care (i.e. follow-up and coordination care) to prevent early readmissions. This study examined whether dementia severity before admission was associated with PAR within 90 days (90-day PAR). This retrospective cohort study was conducted using a Diagnosis Procedure Combination database linked with routinely collected dementia assessment data from a large acute general hospital in Tokyo, Japan. Patients aged 65 or older who were discharged to home or facilities (n=8,910; mean age: 79.8 years, standard deviation: 7.4 years) between July 2016 and September 2018. The dementia severity was classified as normal, slight, moderate, severe dementia based on the Dementia Assessment Sheet for Community-based Integrated Care System 21-items (DASC-21) from the patient or their family at admission. We conducted a multivariable logistic regression adjusted for covariates (sex, age, insurance copayment rate, diagnosis at admission, Charlson Comorbidity Index, unscheduled admission, ICU utilization, surgical treatment, length of hospital stay, discharge place) to examine the association of severity of dementia with 90-day PAR. Among the patients, 225 (2.5%) experienced 90-day PAR. The adjusted odds of 90-day PAR among patients with moderate dementia were 1.571 times (95% confidence interval [CI]: 1.102-2.240) and patients with severe dementia were 2.386 times (95% CI: 1.294-4.398) higher than the odds among patients without dementia. Patients with moderate and severe dementia before admission would be the target with high priority for providing transitional care.

